# The *de novo* genome of the Black-necked Snakefly (*Venustoraphidia nigricollis* Albarda, 1891): A resource to study the evolution of living fossils

**DOI:** 10.1093/jhered/esad074

**Published:** 2023-11-21

**Authors:** Magnus Wolf, Carola Greve, Tilman Schell, Axel Janke, Thomas Schmitt, Steffen U Pauls, Horst Aspöck, Ulrike Aspöck

**Affiliations:** Senckenberg Biodiversity and Climate Research Centre (BiK-F), Frankfurt am Main, Germany; Institute for Ecology, Evolution and Diversity, Goethe University, Frankfurt am Main, Germany; Senckenberg Biodiversity and Climate Research Centre (BiK-F), Frankfurt am Main, Germany; LOEWE-Centre for Translational Biodiversity Genomics (LOEWE-TBG), Frankfurt am Main, Germany; Senckenberg Biodiversity and Climate Research Centre (BiK-F), Frankfurt am Main, Germany; LOEWE-Centre for Translational Biodiversity Genomics (LOEWE-TBG), Frankfurt am Main, Germany; Senckenberg Biodiversity and Climate Research Centre (BiK-F), Frankfurt am Main, Germany; Institute for Ecology, Evolution and Diversity, Goethe University, Frankfurt am Main, Germany; LOEWE-Centre for Translational Biodiversity Genomics (LOEWE-TBG), Frankfurt am Main, Germany; Senckenberg German Entomological Institute, Müncheberg, Germany; Entomology and Biogeography, Faculty of Science, Institute of Biochemistry and Biology, University of Potsdam, Potsdam, Germany; LOEWE-Centre for Translational Biodiversity Genomics (LOEWE-TBG), Frankfurt am Main, Germany; Senckenberg Research Institute and Natural History Museum Frankfurt, Frankfurt am Main, Germany; Institute of Insects Biotechnology, Justus-Liebig-University Giessen, Giessen, Germany; Institute of Specific Prophylaxis and Tropical Medicine, Medical Parasitology, Medical University of Vienna (MUW), Vienna, Austria; Department of Evolutionary Biology, University of Vienna, Vienna, Austria; Department of Entomology, Natural History Museum Vienna, Vienna, Austria

**Keywords:** *de novo* genome assembly, genome annotation, living fossil, phylogenetics, phylogenomics, Neuropterida, Raphidiidae, Raphidioptera

## Abstract

Snakeflies (Raphidioptera) are the smallest order of holometabolous insects that have kept their distinct and name-giving appearance since the Mesozoic, probably since the Jurassic, and possibly even since their emergence in the Carboniferous, more than 300 million years ago. Despite their interesting nature and numerous publications on their morphology, taxonomy, systematics, and biogeography, snakeflies have never received much attention from the general public, and only a few studies were devoted to their molecular biology. Due to this lack of molecular data, it is therefore unknown, if the conserved morphological nature of these living fossils translates to conserved genomic structures. Here, we present the first genome of the species and of the entire order of Raphidioptera. The final genome assembly has a total length of 669 Mbp and reached a high continuity with an N50 of 5.07 Mbp. Further quality controls also indicate a high completeness and no meaningful contamination. The newly generated data was used in a large-scaled phylogenetic analysis of snakeflies using shared orthologous sequences. Quartet score and gene concordance analyses revealed high amounts of conflicting signals within this group that might speak for substantial incomplete lineage sorting and introgression after their presumed re-radiation after the asteroid impact 66 million years ago. Overall, this reference genome will be a door-opening dataset for many future research applications, and we demonstrated its utility in a phylogenetic analysis that provides new insights into the evolution of this group of living fossils.

## Introduction

**Fig. 1.**
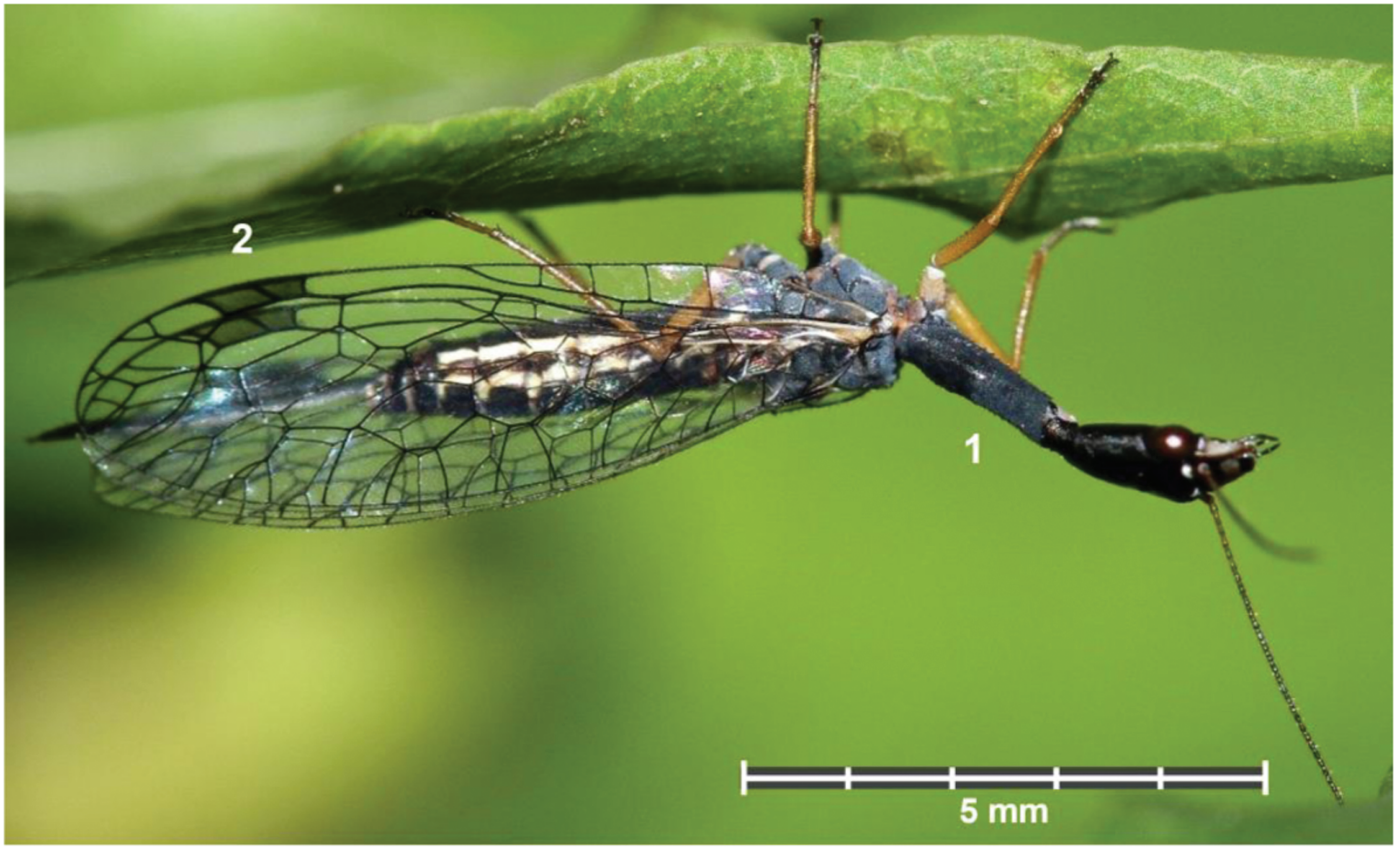
*Venustoraphidia nigricollis* (Albarda 1891), female. Austria inferior, above Klosterneuburg, 48°31ʹN″/16°32ʹE″, 320 m, 19 May 2013, H. & U. Aspöck leg. (Photo H. Bruckner). The species can be differentiated from other snakeflies in Central Europe by the characteristic, totally black pronotum giving the impression of a “black neck” (1), the dark ochre pterostigma in the distal part of both wings (2), and the overall smaller size.

Snakeflies (Raphidioptera), also known as camel-neck-flies, have a rather remarkable appearance, and they owe their name to the upraised head and prothorax resembling a snake-like shape (Fig. 1). They represent one of the three orders of the Neuropterida and are sister group of the Megaloptera and Neuroptera (Aspöck and Aspöck 2005, 2022). To date, 252 extant species of snakeflies have been described, which belong to two families, Raphidiidae (206 species, 27 genera) and Inocelliidae (46 species, 6 genera). They thus represent the smallest order of holometabolous insects.

As with many species-poor insect orders, Raphidioptera resembles a taxonomically ancient group that first appeared during the Carboniferous period, over 300 million years ago (Mya) (Haring et al. 2011). Today, they are restricted to arboreal habitats in the northern hemisphere, but during the Mesozoic era (252 to 66 Mya), they had a much larger distribution, also occurring in the southern hemisphere and in tropical regions (Aspöck 1998; Aspöck et al. 2012). The impact of the asteroid 66 Mya correlates with the near extinction of the Raphidioptera, and those that survived that were already adapted to the following low temperatures as indicated by their previous wide distribution in tropical areas and the dependency of the larvae of extant Raphidioptera on cold temperatures (Aspöck 2012; Aspöck et al. 2019). However, the many Mesozoic snakefly fossils show striking similarities with extant snakeflies, making the Raphidioptera “living fossils” par excellence (Aspöck et al. 2012).

In general, snakeflies are relatively small insects and mostly measure less than 20 mm in body length (Aspöck et al. 1991). Their most characteristic features are an elongated prothorax, hyaline wings, net-like venation, a colored pterostigma as well as the long ovipositor (Fig. 1). Among the smallest snakeflies with a forewing length of 6 mm is a Raphidiid from Central Europe (*Venustoraphidia nigricollis*), and the largest known species is an Inocelliid from China with a forewing length of 18 mm. The identification to date is partly based on external characters and partly on the morphology of male genital sclerites (Aspöck et al. 1991). Adult Raphidiidae feeds on small arthropods, and adult Inocelliidae take up pollen occasionally.

The Black-necked Snakefly (*V. nigricollis*Albarda 1891), is a widespread European snakefly species that was recorded for all Central European countries, eastern Europe, the Balkan Peninsula, the Apennine Peninsula, and southwestern France. Biogeographically, the species is assumed to be polycentric and consequently should have survived glacial periods in several Mediterranean refugia (Aspöck et al. 1991; Aspöck and Aspöck 2022). Among the 16 snakefly species so far known for Central Europe, *V. nigricollis* is the smallest, with a forewing length of 6 to 8.5 mm. With its elongated, totally black pronotum and dark ochre pterostigma, it can be identified with the naked eye (Fig. 1, Aspöck and Aspöck 2022).

For a long time, *V. nigricollis* was considered one of the rarest snakeflies, until it was realized that the imagines mainly live in the canopy layer of deciduous oak and fruit trees (Gruppe and Schubert 2001), a microhabitat strongly underrepresented in entomological surveys. The larvae are corticolous living in crevices of the bark of deciduous as well as coniferous trees. These predatory larvae can play a role in the control of fruit tree pests (Aspöck and Aspöck 2022). They overwinter twice and pupate after hibernation in the third year. The adults appear in May and June. Thus, the development from egg to imago takes 2 yr (Aspöck 2002; Aspöck and Aspöck 2022).

The systematic position of *Venustoraphidia* was still debated in the 1990s, and the genus seemed to be systematically isolated from others (Aspöck et al. 1991). In a first phylogenetic study, a clade was detected that included *Xanthostigma* and *Venustoraphidia* as well as some other genera, that is, the *Puncha* clade (Haring et al. 2011). A phylogenomic analysis based on transcriptomes established *Venustoraphidia* as the sister group of *Puncha*, which together are the sister group of *Xanthostigma* (Vasilikopoulos et al. 2020). This confirmed the before erected *Puncha* clade. As pointed out by Vasilikopoulos et al. (2020), however, phylogenetic conflicts might be abundant in this clade and the entirety of Raphidiidae. Given the broad scope of Vasilikopoulos et al. (2020), which assessed Neuropterida as a whole, these phylogenetic conflicts were never described in detail.

In order to increase the awareness of this conspicuous and notable insect species, and to provide an important resource for further genomic studies of these living fossils, we sequenced and assembled a high-quality reference genome using long-read sequencing technology (PacBio). The obtained assembly and annotation were further used to generate a phylogenetic tree of the Raphidiidae, and to characterize the frequency of phylogenetic conflicts at different positions within the evolution of snakeflies.

## Materials and methods

### Sample origin

One adult male of *V. nigricollis* from Eichkogel, Austria inferior, 48°3ʹ45″N 16°17ʹ33″E, 355 m, 22 May 2022, collected and identified by H. & U. Aspöck and preserved in 96% ethanol. For reference, multiple other individuals from the same area were deposited (ref. Nos. e.g. V. n. m 23/1, V. n. m 23/2, V. n. f 23/3) in the Natural History Museum Vienna (Naturhistorisches Museum Wien).

### DNA isolation and library preparation

Genomic DNA was extracted from thorax tissue according to the protocol of Sambrook and Russell (2001). DNA concentration and DNA fragment length were assessed using the Qubit dsDNA BR Assay kit on the Qubit Fluorometer (Thermo Fisher Scientific) and the Genomic DNA Screen Tape on the Agilent 2200 TapeStation system (Agilent Technologies), respectively. The SMRTbell library was constructed following the instructions of the SMRTbell Express Prep kit v2.0 with Low DNA Input Protocol. Total input DNA was approximately 450 ng. One SMRT cell sequencing run was performed in CCS mode using the Sequel System IIe with the Sequel II Binding kit 3.2 (Pacific Biosciences, Menlo Park, California). The library was loaded at an on-plate concentration of 80 pM using diffusion loading.

### Assembly

A table listing all software, programs, and parameter used to produce shown results can be found in Supplementary Table S1. HiFi reads were retrieved from the raw subreads.bam file using a pipeline consisting of PacBio’s ccs 6.4.0 and actc 0.3.1 as well as DeepConsensus 0.3.1 (Baid et al. 2023). All HiFi reads were assembled using hifiasm 0.16.1 (Cheng et al. 2021, 2022). Raw primary contigs were screened for contamination using blobtools 1.1.1 (Laetsch and Blaxter 2017), and taxonomic assignments of primary contigs were based on sequence similarity to the nt database determined by blastn 2.12.0+ and an e-value cutoff set to 1e-25 (Camacho et al. 2009). Coverage distribution was obtained by mapping all HiFi reads to the assembly using backmap 0.5 (https://github.com/schellt/backmap) in combination with minimap 2.24 (Li 2018), and samtools 1.15 (Danecek et al. 2021).

The mitochondrial genome was obtained with MitoHiFi 2.2 (DOI 10.5281/zenodo.6451619) using the raw primary contigs, the mitochondrial genome *of Mongoloraphidia harmandi* (NC_013251.1) as a reference, under the mitochondrial invertebrate genetic code (setting “5”). The resulting mt genome sequence was searched with blastn in the raw primary contigs of hifiasm.

After contamination screening and the generation of a mitochondrial genome, sequences were removed from the primary contigs of the genome assembly that hifiasm flagged as circular. So were sequences that were taxonomically assigned as Mollusca, Annelida, or Chordata during the blobtools analysis, as well as those that were aligned to the mitochondrial genome sequence with more than 85% of their total length.

The filtered contigs were then polished using all HiFi reads. This was done by first mapping the HiFi reads to the filtered contigs using minimap 2.24. The mapping results were sorted by coordinates using samtools 1.15. Duplicates were removed using picard 2.26.10 MarkDuplicates (https://github.com/broadinstitute/picard). The assembly fasta file and the duplicate filtered bam file were indexed with samtools faidx and samtools index, respectively. Variants were identified using DeepVariant 1.2 (https://github.com/google/deepvariant) with. Resulting heterozygous variants were filtered out with bcftools 1.15 (Danecek et al. 2021) using the command “view.” The compressed vcf file was then indexed using tabix from HTSlib 1.15 (Bonfield et al. 2021). Finally, bcftools consensus was used to generate the polished contigs from the filtered hifiasm contigs and the filtered variant set. A depiction of the coverage distribution of the assembly after polishing can be found in Supplementary Fig. S1.

### Quality checks

Assembly accuracy was estimated with Merqury 1.3 (Rhie et al. 2020) in combination with its dependencies Meryl 1.3 (https://github.com/marbl/meryl), samtools 1.15, bedtools 2.30.0 (Quinlan and Hall 2010), and R 4.0.3. To do so, all HiFi reads were used to create a 21-mer database against which assemblies were compared. To assess completeness regarding single-copy orthologs, BUSCO 5.4.3 (Manni et al. 2021) was run with the insecta_odb10 set. Basic assembly statistics were calculated and summarized together with the BUSCO results in a snail plot with blobtoolkit 4.1.4 (Challis et al. 2020). To infer the fraction of assembled reads and the coverage distribution, backmap.pl 0.5 in combination with above-mentioned tools and Qualimap 2.2.1 (Okonechnikov et al. 2016), bedtools 2.30.0, R 4.0.3, and MultiQC 1.12 (Ewels et al. 2016) was applied. To infer genome size, ModEst (Pfenninger et al. 2022) was used as implemented in backmap.pl.

### Repeat and gene annotation

We identified repeats specific to *V. nigricollis* using RepeatModeler 2.0.1 (Flynn et al. 2020) in combination with RepeatMasker 4.1.0 (www.repeatmasker.org/RepeatMasker/), RECON 1.08 (Bao and Eddy 2002), RepeatScout 1.0.6 (Price et al. 2005), Tandem Repeats Finder 4.10 (Benson 1999), and RMBlast 2.11.0+ (www.repeatmasker.org/rmblast/). Resulting repeat families were combined with all Hexapoda repeat sequences from RepBase release 27.06 (Bao et al. 2015) and used as input for RepeatMasker 4.1.2. A repeat landscape was constructed based on the RepeatMasker output by running calcDivergenceFromAlign and createRepeatLandscape from the RepeatMasker tool collection with default parameters.

The soft-masked genome assembly was used for gene annotation as implemented in the BRAKER3 pipeline (Buchfink et al. 2015; Hoff et al. 2016; Kovaka et al. 2019; Brůna et al. 2021; Bruna et al. 2023; Gabriel et al. 2023). This approach combines a *de novo* gene calling, transcriptome-based gene annotation using the transcriptome of *V. nigricollis* (Vasilikopoulos et al. 2020), and a homology-based gene annotation. For protein references, we combined the Arthropoda-specific protein collection from OrthoDB (arthropoda_odb10, retrieved: 19 Jan 2023) following the recommendations in the BRAKER user guide (github.com/Gaius-Augustus/BRAKER). The resulting set of proteins was tested for completeness using BUSCO v.5.4.75.3.1 (Manni et al. 2021) in “protein mode” and run against the insect-specific set of core genes. Functional annotation was done using InterProScan v5 (Jones et al. 2014).

### Phylogenetic analysis

Phylogenetic reconstruction was performed using the BUSCO-to-Phylogeny wrapper function (Schneider et al. 2021). The applied code is available on github.com (mag-wolf/BUSCO-to-Phylogeny). Publicly available RNA data (Supplementary Table S2) of other Raphidioptera species were downloaded from NCBI SRA, and short reads were assembled into transcriptomes using Trinity v2.8.5 (Grabherr et al. 2011) with default parameters. The resulting transcriptomes, as well as the genome assembly constructed here, were annotated using the BUSCO v5.4.3 (Manni et al. 2021) function in short mode and restricted to the insecta_odb10 dataset of OrthoDB (Kriventseva et al. 2019). We extracted single-copy orthologous sequences (SCOS) with no more than 25% missing species and orthologous sequences were aligned using Mafft v7.475 (Katoh and Standley 2013) with 1,000 iterative refinements. Alignments were trimmed using ClipKit v1.1.3 (Steenwyk et al. 2020) in the “kpic-smart-gap” mode to allow for an additional smart-gap-based trimming. Based on the trimmed alignments, gene trees were constructed using IQtree v2.1.2 (Minh et al. 2020) with 1,000 bootstrap replications each. We further filtered gene trees and alignments based on the maximum likelihood genetic distance calculated by IQtree. To do this, we removed orthologs in the 5% and 95% quantiles to avoid misalignments and sequences with too little information for a meaningful tree construction. All alignments were then concatenated using FASconCAT-G v1.04 (Kück and Longo 2014), and an overall tree was constructed using IQtree with 1,000 bootstrap replicates. To estimate the genealogical concordance in this phylogenetic dataset, IQtree was further used to calculate the gene concordance factor (gCF) and site concordance factor (sCF) using the “--gcf” and “--scf” parameters, respectively. Astral-III v5.7.3 (Zhang et al. 2018) was used to create a consensus tree based on all individual gene trees, which also performed a quartet score (QS) calculation to assess the level of genetic conflict within the dataset. Respective statistics can be found in Supplementary Table S3. To test whether short branches result in more genetic conflicts, we performed a Pearson correlation test in R (Supplementary Fig. S2).

## Results

### Genome properties

**Fig. 2.**
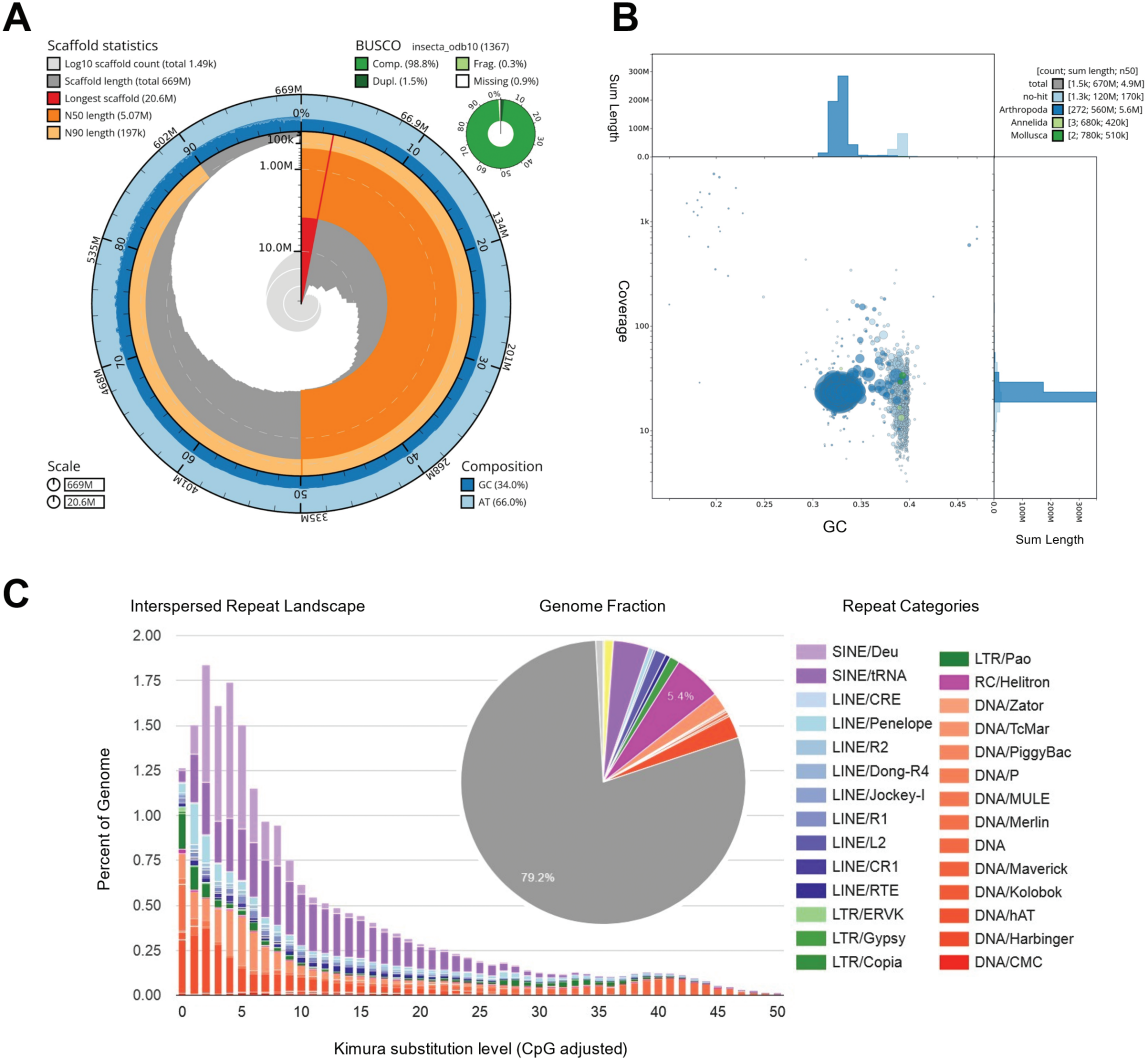
Plots summarizing different properties of the newly compiled reference genome for *Venustoraphidia nigricollis*. A) Snail plot depicting contig statistics in the innermost circle with colors indicating the longest contig, N50, and N90, respectively. GC and AT composition are indicated in the outer circle with colors indicating the amounts of both categories, respectively. BUSCO completeness statistics are showcased in the small green circle with colors indicating the different categories assessed by BUSCO. B) Blobplot depiction of coverage distribution, GC content, and NCBI BLAST hits of PacBio reads before contamination filtering. Reads represented by green circles were removed prior to genome assembly. C) Repeat landscape of the newly compiled genome assembly. Colors represent types of repetitive regions. Gray areas indicate unclassified types of repetitive regions.

Sequencing and HiFi calling resulted in a total of 2,379,419 reads with a total length of 13,493,408,460 bp and a read N50 of 6,109 bp. The genome was assembled to a total size of 669,157,981 bp and consists of 1,485 contigs with an N50 of 5,066,507 bp and an L50 of 37 (Fig. 2A). The longest contig was assembled to a length of 20,626,596 bp. The GC content was 34%, and no gaps were introduced to the assembly. BUSCO completeness was overall high with 98.8% complete orthologous sequences found within the assembly.

Contiguity increased slightly after the removal of contaminations. A total of 6,914 variants were applied during polishing, which increased accuracy as shown by Merqury as error rate and QV estimates. Completeness with respect to single-copy orthologs was generally unaffected by removing contamination or by polishing. Mapping rates are consistently high, and the coverage distribution appears unimodal with a small hump toward lower coverage. ModEst genome size estimates based on the mode of the mapping coverage distribution resulted in 636 Mb, which is only 5% less than the final assembly length. For comparison see Supplementary Table S4. Contamination screening revealed only small traces of contamination in the form of different taxonomic assignments (Fig. 2B).

In total, 64.6% of the genome were masked as repetitive region with RepeatMasker. Most of these repeats were unclassified (42.9%), and retroelements (8.8%) were more abundant than DNA transposons (6.5%). For further detail see the repeat landscape graph (Fig. 2C). Genome annotation resulted in 14,126 potential genes of which 969 (6.9%) were functionally characterized using InterProScan. BUSCO completeness statistics of the protein sequences were also high, with a total of 96% found orthologs, of which 14.6% were duplicated.

### Phylogenetics

The phylogenetic tree features 381 high-quality SCOS shared between 15 species of Raphidiidae, including the newly presented reference genome of *V. nigricollis*. Based on this set of genes, a 138 kb long concatenated alignment was compiled that contained 13,659 informative sites. Using this alignment, a maximum likelihood tree was constructed along with 1,000 bootstrap replications, and branches with less than 50% support were collapsed in the final tree. *Inocellia crassicornis* (Inocelliidae) was included as an outgroup. The individual of *V. nigricollis* sequenced in this study was placed together with the RNA data of the *V. nigricollis* individual sequenced by Vasilikopoulos et al. (2020). The tree generally agrees with Vasilikopoulos et al. (2020) in almost all branches not collapsed in the final tree as it relies on the same underlying RNA data, but it also supports the previously established *Puncha* clade based on nuclear and mitochondrial marker sequences (Haring et al. 2011). The only conflict between this and other trees was the grouping of *Raphidia mediterranea* and *Turcoraphidia amara*, which was supported with a lower bootstrap value of 84% and 13.3% gCF in the tree presented herein. The QS and gene concordance analyses revealed a high number of alternative topologies within the set of gene trees over all measured branches. Branch 1, splitting *Monogoloraphidia sororcula* from the majority of Raphidiidae, showed a higher frequency of one of both possible alternative topologies. Branch 2, which divides the group into two clades, had an excess of alternative topologies compared with the topology constructed using the overall maximum likelihood approach. The correlation test between discordance factors (QS, gCF, and sCF) and branch lengths revealed a general correlation trend between short branches and higher amounts of genetic conflicts. In case of the side concordance factor, this correlation was significant (*R* = 0.72, *P* = 0.045). For details see Supplementary Fig. S2 and Supplementary Table S3. We further noted that Branch 1 appears as an outlier in the correlation analysis because this branch features a longer branch length in relation to its found genetic conflicts.

## Discussion

### High-quality reference genome

To our knowledge, the presented genome of *V. nigricollis* (Black-necked Snakefly) is the first *de novo* reference genome within Raphidioptera and is one of only a few available genomes within the superorder Neuropterida. Due to the use of long-read sequencing technology, we reached a high continuity that is only topped by genome assemblies using additional Hi-C technology, as in Neuropterida, for example, the green lacewing species *Chrysopa pallens* (Wang et al. 2022). We further expect the genome assembly to be highly complete due to both the conducted genome size estimation and the BUSCO gene completeness analysis. Contamination checks using Blobtools also revealed no major contamination from symbionts or bacteria, as often cannot be avoided when sequencing small animals. The genome featured a high amount of repetitive regions, and the high number of unclassified calls indicate the need for manual curation or a high amount of far unknown types of repeats. The annotated genes using *de novo* gene calling, RNA and protein evidence also resulted in high completeness values, although some duplications were noted in genes belonging to the core set of insect orthologs.

A high-quality reference genome is a valuable tool to study genetic features potentially related to “living fossils.” Genome conservation, expectable in a lineage of organisms with long-lasting phenotype conservation, can, for example, be studied as genome synteny which may be indicative for genome re-structuring processes or the lack thereof (Mathers et al. 2021; Van Dam et al. 2021; Winter et al. 2022). These processes may be largely influenced by transposable elements, and comparing the presented landscape to related species may provide important insights about the dominant type of transposable element as done for other lineages commonly referred to as “living fossils” (Guan et al. 2016; Gemmell et al. 2020; Huang et al. 2022). Other related genome characteristics that could be studied are, for example, gene conservation (Guan et al. 2016; Huang et al. 2022; Van Dam et al. 2021), given their direct influence on the phenotype, or the demographic history (Gemmell et al. 2020), because lower population sizes over a longer period of time usually lead to a lowered genetic diversity due to increased drift and inbreeding (Teixeira and Huber 2021). The high-quality genome of the Black-necked Snakefly represents a first step in this field as now many more members of the order Raphidioptera can be sequenced and compared economically using short-read technology.

### Phylogeny of the family Raphidiidae

**Fig. 3.**
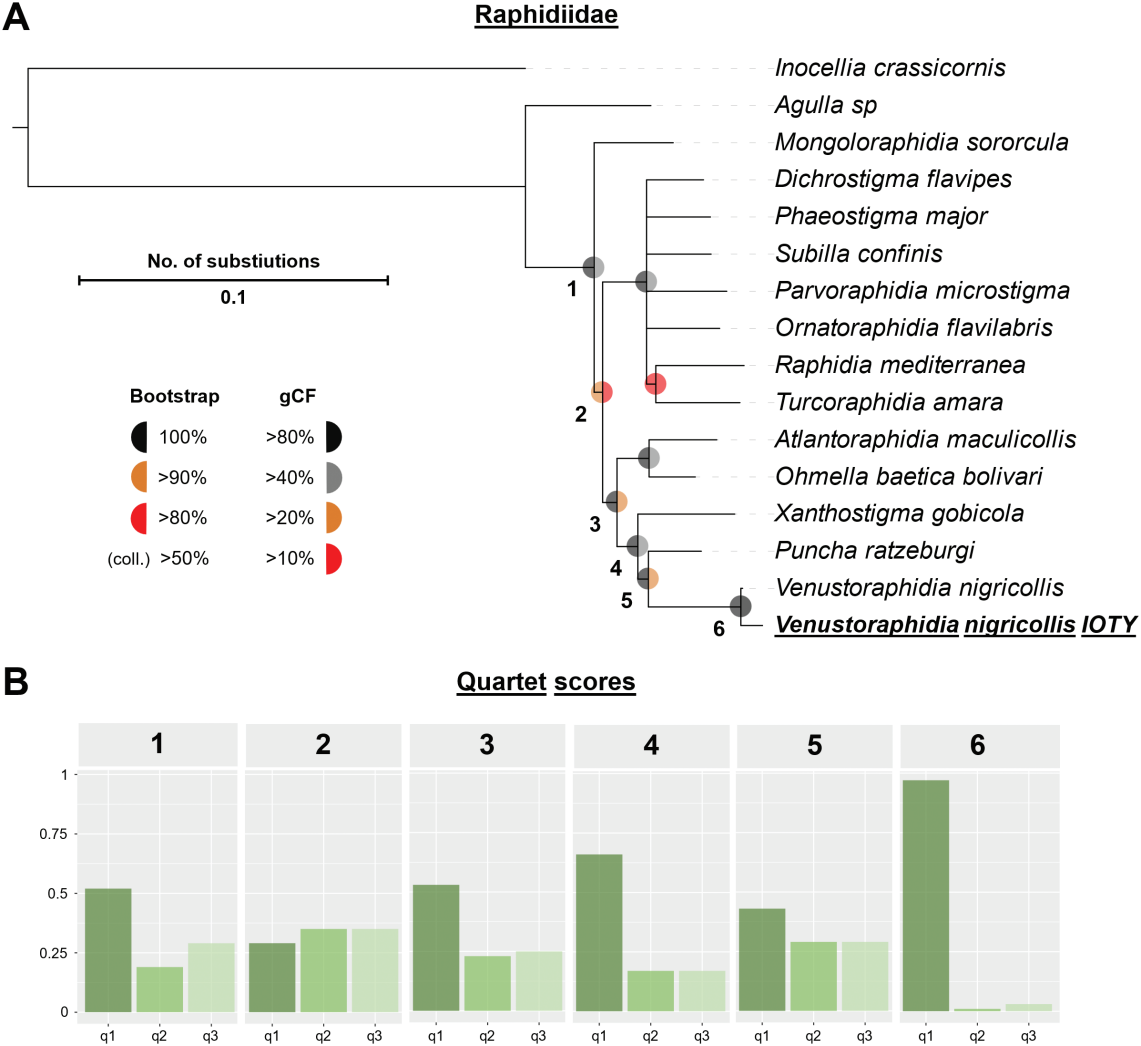
Phylogenetic and quartet score analysis of the Raphidiidae, including the newly compiled reference genome of *V. nigricollis*. A) The maximum likelihood tree was constructed based on a concatenated alignment of 381 high-quality SCOS identified by running BUSCO on transcriptomes published in Vasilikopoulos et al. (2020) and the new genome of *V. nigricollis*. Bootstrap values and gene concordance factors are indicated as a half of a respective colored dot. Branches with less than 50% support were collapsed. B) Quartet score analysis depicts high frequencies of phylogenetic conflicts between the set of SCOS. Branch 1, including all Raphidiidae except *Agulla* sp. showed an excess of one alternative topology (q3). Branch 2, including all residual Raphidiidae except *Mongoloraphidia sororcula* and dividing the group into two major clades, indicates exceptionally even and more frequent occurrences of alternative topologies. Overall, the tree largely coincides with Vasilikopoulos et al. (2020) but showcases the high amount of hidden conflicts that were not discussed so far.

The phylogenetic analysis of the family Raphidiidae showcased the high degree of phylogenetic conflict within this group of insects due to a high frequency of genes that support diverging topologies from the inferred species tree. Apart from potential technical artifacts, phylogenetic conflicts can result from two different phenomena: incomplete lineage sorting (ILS) and introgression (Hibbins and Hahn 2022). Although the Raphidioptera emerged in the late Carboniferous (~320 Mya, Vasilikopoulos et al. 2020) and fossil records from the Mesozoic revealed a surprising similarity to recent snakeflies (Willmann 1994; Jepson and Jarzembowski 2008; Liu et al. 2016), modern species can be traced back to two lineages that adapted to a colder climate that followed the impact of the asteroid 66 Mya (Aspöck et al. 2012). In line with this, molecular clock estimates indicate that the evolution of modern snakefly lineages has taken place within a relatively short period of ~10 million years within the Paleogene (Vasilikopoulos et al. 2020). Such a comparatively rapid divergence could result in a high amount of ILS which is further supported by the general correlation trend between genetic conflicts and branch length (Supplementary Fig. S2, Arcila et al. 2021). This would also explain the conflicting phylogenetic results in previous studies (Haring et al. 2011; Vasilikopoulos et al. 2020), since results might strongly vary depending on the included loci. The neutral MSC model predicts, that ILS occurs at random and with predictable, roughly equal frequencies of alternative topologies (Hibbins and Hahn 2022). This was, however, not the case in two of the analyzed branches (branch 1 and 2 in Fig. 3) since branch 1 featured a higher amount of topology q3 compared with q2, while branch 2 showed a lower frequency of the topology constructed in the concatenated matrix tree (q1) compared with the two alternative frequencies q2 and q3. Such discrepancies from the MSC model could instead be explained by potential introgression events (e.g. Wolf et al. 2023), but further testing is necessary to identify molecular traces of, for example, hybridization. At least some examples of successful hybridization of different species of Raphidioptera are known or at least possible (Aspöck et al. 1991). Deciphering the apparent conflict between the overall morphological conservation across different snakeflies and their relatively recent radiation will be an important task for further research. The compiled high-quality reference genome will play a fundamental role in different analyses as outlined above.

## Supplementary Material

esad074_suppl_Supplementary_Materials

## Data Availability

The *de novo* genome assembly was uploaded to NCBI under the BioProject ID: PRJNA996517, the assembly ID: JAVRKA000000000, and the BioSample ID: SAMN36598611. The circular consensus reads generated with the PacBio HiFi data were uploaded to the NCBI SRA repository: SRR26678467. All other data, including the annotation, the set of orthologs, and the set of gene trees were uploaded to a Drayd repository (Wolf et al. 2023) and can be accessed under the: DOI: https://doi.org/10.5061/dryad.kwh70rz9h. All code used to conduct the presented research is already published and cited accordingly.
