# Supplementary material for: The de novo genome of the Black-necked Snakefly (Venustoraphidia nigricollis Albarda, 1891): A resource to study the evolution of living fossils
Source: J Hered. 2023 Nov 21;115(1):112–9. doi: 10.1093/jhered/esad074 (PMC10838129; doi:10.1093/jhered/esad074)
Supplement: esad074_suppl_Supplementary_Materials [file esad074_suppl_supplementary_materials.pdf]

Supplementary Materials for

The *de novo* genome of the Black-necked Snakefly (*Venustoraphidia nigricollis* Albarda, 1891): A resource to study the evolution of living fossils.

Magnus Wolf, Carola Greve, Tilman Schell, Axel Janke, Thomas Schmitt, Steffen Pauls, Horst Aspöck, Ulrike Aspöck

*Corresponding author: Magnus Wolf; Email: Magnus.Wolf@senckenberg.de

This PDF file includes:

Figures S1 to S2

- **Fig. S1:** Coverage distribution after polishing.
- **Fig. S2:** Pearson correlation test between branch lengths and concordance statistics.

Tables S1 to S4

- **Table S1:** Software, programs and functions used in this study.
- **Table S2:** Transcriptomes used for phylogenetic analysis.
- **Table S3:** Branch lengths and genetic discordance statistics of the phylogenetic tee.
- **Table S4:** Assembly statistics before and after polishing.

Supplementary Figures

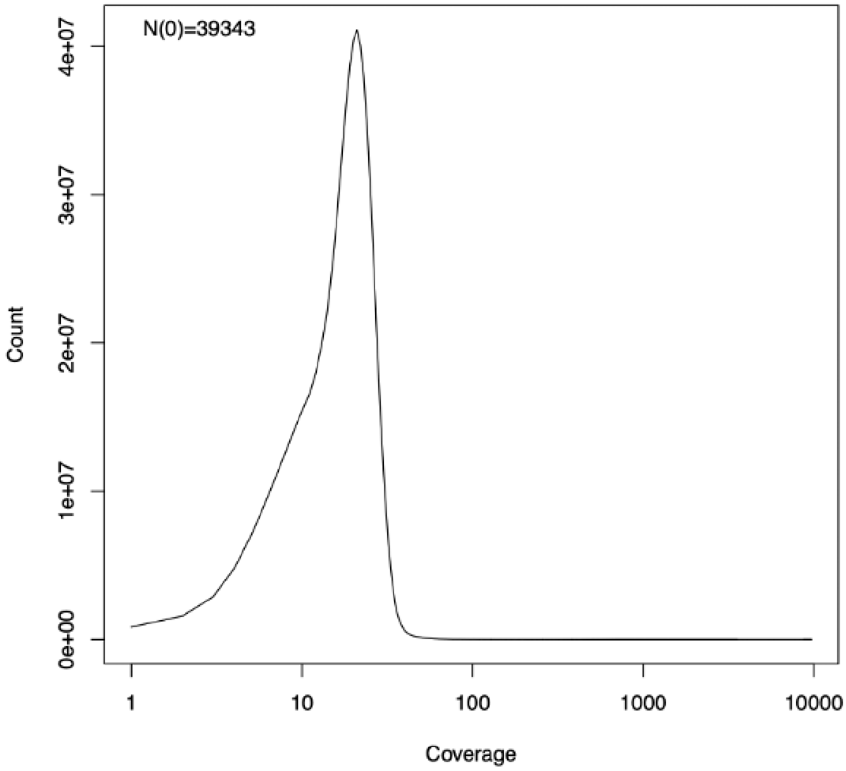

Fig. S1: Coverage distribution after polishing. The coverage is distributed evenly around 21x with a small proportion of lower covered positions.

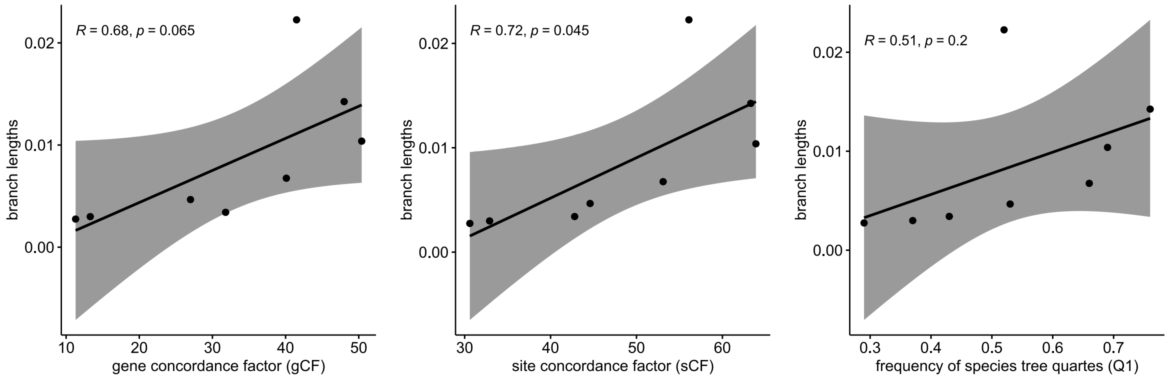

Fig. S2: Pearson correlation test between branch lengths and genetic discordance statistics. The outlier was determined as branch 1 in all statistics (see Figure 3).

Supplementary Tables

Table S1: Software, programs and functions used in this study. Respective citations can be found in the main manuscript. A default usage of a software was noted as “na”.

Software	Version	Description	non-default settings
hifiasm	0.16.1	Cheng et al. 2022	na
blobtools	1.1.1	Laetsch & Blaxter 2017	na
backmap	0.5	(https://github.com/schellt/backmap)	na
minimap	2.24	Li et al.2018	-a -x map-hifi
samtools	1.15	Danecek et al. 2021	-l 9 -O BAM
blastn	2.12.0+	Camacho et al. 2009	-evalue 1e-25
MitoHifi	2.2	DOI 10.5281/zenodo.6451619	-o 5
picard	2.26.10	https://github.com/ broadinstitute/picard	--REMOVE_DUPLICATES
DeepVariant	1.2	https://github.com/google/deepvariant	--model_type=PACBIO
bcftools	1.15	Danecek et al. 2021	-f 'PASS' -i 'GT="1/1" --no-version -Oz
HTSlib	1.15	Bonfield et al. 2021	na
Merqury	1.3	Rhie et al. 2020	na
Meryl	1.3	https://github.com/marbl/meryl	k=21
bedtools	2.30.0	Quinlan & Hall 2010	na
BUSCO	5.4.3	Manni et al. 2021	--short --lineage insecta_odb10
blobtoolkit	4.1.4	Challis et al. 2020	na
Qualimap	2.2.1	Okonechnikov et al. 2016	na
MultiQC	1.12	Ewels et al. 2026	na
RepeatModeler	2.0.1	Flynn et al. 2020	-pa 16 -LTRStruct
RepeatMasker	4.1.0.	www.repeatmasker.org/RepeatMasker/	-xsmall -no_is -e ncbi -pa 16 -s
RECON	1.08	Bao & Eddy 2002	na
RepeatScout	1.0.6	Price et al. 2005	na
Tandem Repeats Finder	4.10	Benson 1999	na
RMBlast	2.11.0+	www.repeatmasker.org/rmblast/	na
calcDivergenceFromAlign	4.1.0.	www.repeatmasker.org/RepeatMasker/	na
createRepeatLandscape	4.1.0.	www.repeatmasker.org/RepeatMasker/	na
BRAKER	3	Gabriel et al. 2023	--gff3
InterProScan	5	Jones et al. 2024	-dp -iprlookup -goterms
BUSCO-to-Phylogeny	2	Schneider et al. 2021	na
Trinity	2.8.5	Grabherr et al. 2011	--trimmomatic
Mafft	7.475	Katoh & Standley 2013	--maxiterate 1000 --localpair
ClipKit	1.1.3	Steenwyk et al. 2020	-kpic-smart-gap
IQtree	2.1.2	Minh et ak. 2020	-bb 1000 -gcf -scf
FASconCAT-GT	1.04	Lück & Longo 2014	-s
Astral-III	5.7.3	Zhang et al. 2018	na

Table S2: Transcriptomes used for phylogenetic analysis. Data published in Vasilikopoulos et al. (2020) (reference in main manuscript).

Species	SRA accession number
Inocellia crassicornis	SRR921607
Agulla sp.	SRR1811836
Mongoloraphidia sororcula	SRR1811882
Dichrostigma flavipes	SRR1811855
Phaeostigma major	SRR1811896
Subilla confinis	SRR1811911
Parvoraphidia microstigma	SRR2774020
Ornatoraphidia flavilabris	SRR1811893
Raphidia mediterranea	SRR1811904
Turcoraphidia amara	SRR1811914
Atlantoraphidia maculicollis	SRR1811838
Ohmella baetica bolivari	SRR1811892
Xanthostigma gobicola	SRR1811918
Puncha ratzeburgi	SRR1811903
Venustoraphidia nigricollis	SRR1811915

Table S3: Branch lengths and concordance statistics of the phylogenetic tee. Branches are numbered after Figure 3. The table includes gene concordance factors (gCF), side concordance factors (sCF), frequencies of alternative quartets (Q1-Q3), branch lengths and bootstrap support values for the preceding (mother) branch.

Branch	gCF	sCF	Q1	Q2	Q3	B. length	BS
**1**	41.5	56.1	0.52	0.19	0.29	0.02226	100
**2**	11.3	30.6	0.29	0.35	0.35	0.00275	91
**3**	27.0	44.6	0.53	0.23	0.25	0.00466	100
**4**	40.1	53.1	0.66	0.17	0.17	0.00675	100
**5**	31.8	42.8	0.43	0.29	0.29	0.00341	100
**6**	94.3	98.9	0.97	0.01	0.03	0.02995	100
**7**	48.0	63.3	0.76	0.13	0.12	0.01425	100
**8**	13.3	32.9	0.37	0.32	0.32	0.00299	84
**9**	50.4	63.9	0.69	0.16	0.15	0.01038	100

Table S4: Assembly statistics before and after polishing.

	Hifiasm primary contigs	After polishing
**Number of contigs**	1,536	1,485
**Total length (bp)**	674,515,966	669,157,981
**N50**	4,941,835	5,066,507
**QV**	58.3208	62.2771
**Error rate**	1.47204e-06	5.91961e-07
**BUSCO**	C:99.0%[S:97.4%,D:1.6%], F:0.3%,M:0.7%,n:1367	C:98.8%[S:97.3%,D:1.5%], F:0.3%,M:0.9%,n:1367
**Mapping rate (%)**	99.96	99.93
**Mapping coverage**	21	21
**Genome size estimate (Mb)**	636.52	635.93
